# The Characteristic “Tunnel Sign” on MRI as a Diagnostic Marker of Tunneling Abscesses in Listeria Rhombencephalitis in a Patient With Multiple Myeloma: A Case Report

**DOI:** 10.7759/cureus.103000

**Published:** 2026-02-04

**Authors:** Madhushan Ranabahu, Nipuna Weerasinghe, Janya Jayawardena, Kishan Dissanayake, Janaka Peiris

**Affiliations:** 1 Medicine, Postgraduate Institute of Medicine, University of Colombo, Colombo, LKA; 2 Neurology, National Hospital Kandy, Kandy, LKA; 3 Radiology, National Hospital Kandy, Kandy, LKA

**Keywords:** ampicillin, chemotherapy, immunocompromised, immunoparesis, ophthalmoplegia

## Abstract

Neurolisteriosis is a rare but potentially serious CNS infection. Diagnosis can be established through bacteriological studies or by identifying characteristic tunneling abscesses in the CNS. Delayed initiation of appropriate antibiotic therapy may result in permanent structural brain damage, disability, or death. We report the case of a 65-year-old woman with multiple myeloma undergoing chemotherapy who presented with an acute febrile illness accompanied by drowsiness, slurred speech, difficulty swallowing, neck stiffness, and complex ophthalmoplegia. MRI of the brain demonstrated characteristic findings of the “tunnel sign,” suggestive of a tunneling abscess in *Listeria* rhombencephalitis. The diagnosis was made based on radiological features alone, in the absence of microbiological confirmation. With prompt initiation of IV ampicillin, she demonstrated marked neurological recovery, with some residual deficits.

## Introduction

*Listeria monocytogenes* is a gram-positive intracellular bacillus transmitted to humans through food contamination. Listeriosis is a foodborne infection caused by the consumption of unheated, processed, refrigerated, or canned meat and fish products, as well as unpasteurized dairy products [1,2]. *Listeria *is a neurotropic bacterium that primarily affects the CNS, causing neurolisteriosis. Neurolisteriosis is common in neonates, pregnant women, the elderly, and immunocompromised patients [3]. CNS involvement in neurolisteriosis can manifest as meningitis, encephalitis, and, rarely, abscess formation [4]. In immunocompromised patients, neurolisteriosis has a high case-fatality rate of approximately 30% [5]. Diagnosis can be established by isolating the bacteria from CSF using Gram staining, culture, or PCR. CNS imaging is also essential, as contrast-enhanced MRI can reveal characteristic tunneling abscesses in 1-10% of patients [3,6].

## Case presentation

**Figure 1:**
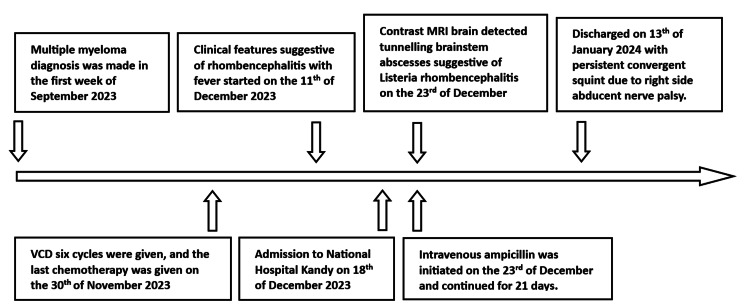
Timeline illustrating the chronological progression of the patient’s illness

A 65-year-old female tailor was diagnosed with multiple myeloma during the first week of September 2023. She received six cycles of VCD (bortezomib, cyclophosphamide, and dexamethasone) for treatment. Her last chemotherapy session was on November 30, 2023. Following this, she experienced generalized ill health for 10 days. Subsequently, over seven days, she developed fever, headache, nausea, vomiting, loss of appetite, diplopia, reduced vision, slurred speech, and difficulty swallowing (Figure 1).

She was admitted to National Hospital Kandy on December 18, 2023. Physical examination revealed an ill-appearing patient with a temperature of 38.8°C, a blood pressure of 130/80 mmHg, a regular pulse of 86 beats per minute, and a respiratory rate of 18 breaths per minute. Precordial, lung, and abdominal examinations were normal. Detailed neurological examination showed a Glasgow Coma Scale score of 13/15 (eye 3, verbal 4, motor 6), neck stiffness, right eye deviation medially with complex ophthalmoplegia, and pupillary size of 3 mm bilaterally with sluggish direct and consensual light reflexes. Motor power was 3/5 in all limbs with normal reflexes, and plantar responses were downgoing bilaterally.

An initial clinical diagnosis of meningoencephalitis was made in this elderly patient with multiple myeloma undergoing chemotherapy. Empirical IV ceftriaxone 2 g every 12 hours was initiated after obtaining a peripheral blood culture on December 18. A diagnostic lumbar puncture was performed on December 20, 2023. CSF analysis revealed lymphocytic pleocytosis with low glucose (Table 1).

**Table 1:** Laboratory findings of the patient IHA, indirect hemagglutination assay; TB PCR, tuberculosis PCR; VDRL, Venereal Disease Research Laboratory

Parameter	Result	Reference range
White blood count	10.09 (neutrophils 76%)	4-10 × 10³/µL
Hemoglobin	11.7	12-15 g/dL
Platelets	245	150-450 × 10³/µL
Corrected calcium	2.02	2.15-2.65 mmol/L
Serum creatinine	63	60-120 µmol/L
Sodium	134	135-148 mmol/L
Aspartate transaminase	17	10-35 U/L
Alanine transaminase	13	10-40 U/L
Serum albumin	37	35-52 g/L
Serum globulin	26	25-35 g/L
C-reactive protein	32	0.5-5.0 mg/dL
Erythrocyte sedimentation rate	50	<30 mm/h
Blood culture	Negative	-
Mantoux test	Negative	-
HIV screening	Negative	-
IHA for melioidosis	Negative	-
CSF cryptococcal antigen	Negative	-
CSF TB culture	Negative	-
CSF TB PCR	Negative	-
CSF pyogenic culture	Negative (up to 48 hours incubation)	-
CSF VDRL	Nonreactive	-
CSF color	Clear	-
CSF protein	112	15-45 mg/dL
CSF sugar	43 mg/dL	>2/3 of blood glucose
Serum blood sugar	120	72-200 mg/dL
CSF cells	WBC 250/mm³ (lymphocytes 83%)	<5 lymphocytes/mm³
CSF microscopy	Negative	-

**Figure 2:**
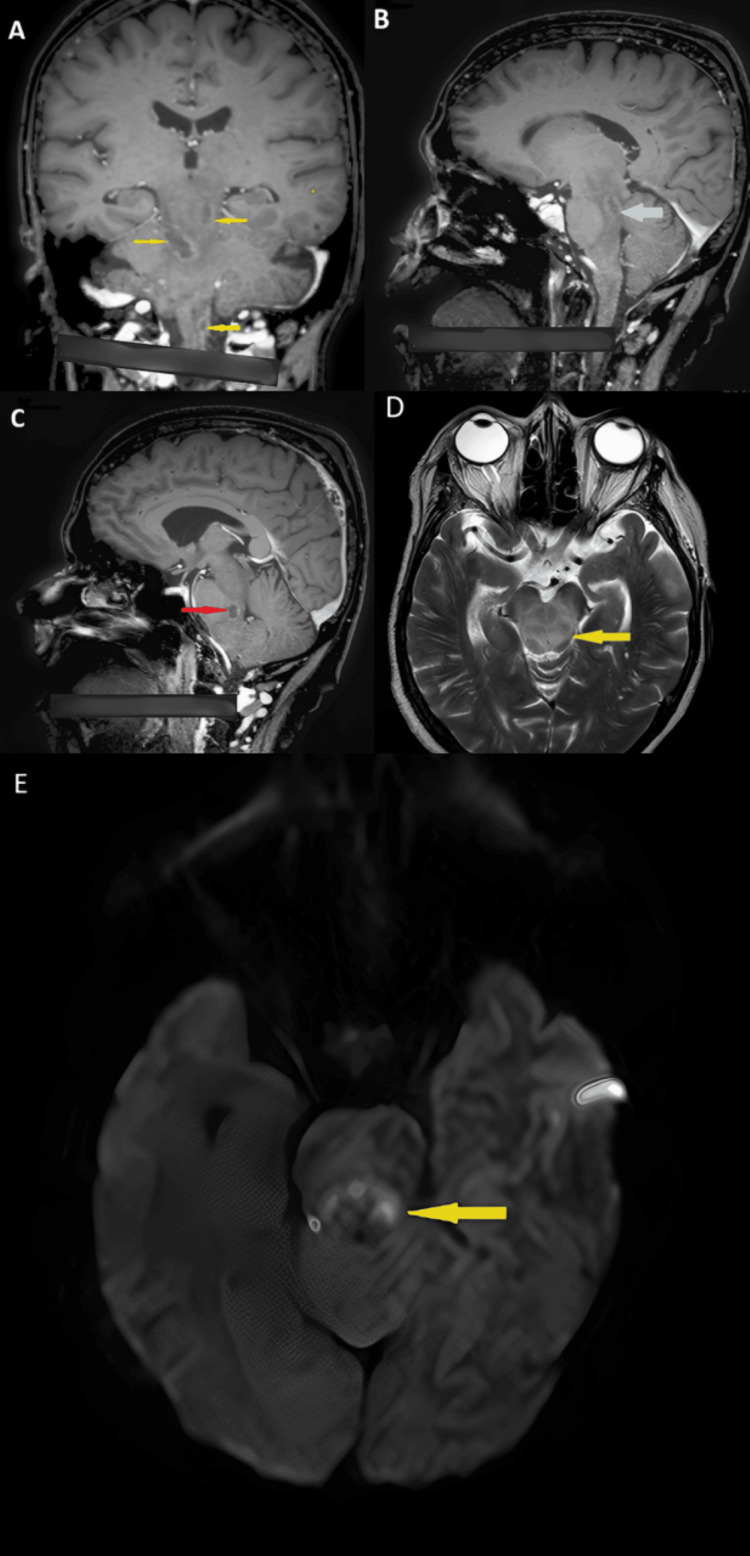
(A) Coronal T1 MR image with contrast of the patient’s brain. Yellow arrows indicate multiple lesions in the midbrain, pons, and upper cervical spinal cord. These lesions demonstrate peripheral ring enhancement with central non-enhancement, typical of tunneling abscesses. Right side is indicated. (B) Sagittal T1 MR image with contrast. Light blue arrow shows a lesion in the upper pontine tegmentum with peripheral ring enhancement and central non-enhancement. (C) Sagittal T1 MR image with contrast. Red arrow shows a lesion in the lower pontine tegmentum with central low intensity and peripheral ring enhancement. (D) Axial T2 MR image. Green arrow indicates midbrain hyperintensity consistent with edema. (E) Axial diffusion-weighted MR image. Yellow arrow demonstrates diffusion restriction within small ring-enhancing lesions, consistent with microabscesses.

MRI of the brain with contrast was performed on December 23, 2023, and demonstrated characteristic findings of brainstem abscesses with the “tunnel sign,” consistent with *Listeria *rhombencephalitis (Figure 2A-2E).

IV ampicillin 2 g was administered every four hours starting on December 23, 2023, and continued for 21 days. Ceftriaxone was discontinued after 10 days. All neurological deficits improved following the ampicillin course, except for a persistent convergent squint due to right lateral rectus weakness from right-sided abducent nerve palsy. The patient was discharged from the hospital on January 13, 2024, with the convergent squint remaining.

## Discussion

The patient was a late middle-aged female with recently diagnosed multiple myeloma undergoing chemotherapy. Multiple myeloma causes immunoparesis due to an excess of uninvolved immunoglobulins and suppression of normal immunoglobulin production. In addition, the patient received six cycles of VCD chemotherapy for multiple myeloma, which further aggravated her immunocompromised status [7].

*L. monocytogenes *spreads into the CNS via either hematogenous dissemination from the gastrointestinal tract or retrograde axonal transport. Interestingly, retrograde axonal spread typically occurs along the trigeminal nerve to the brainstem [4,6]. When *L. monocytogenes* enters the brain white matter, it spreads along axons, causing tunneling abscesses along the pathways of spread. CNS tunneling abscess formation is characteristic of neurolisteriosis [2,6]. However, tunneling abscesses in the CNS can also occur in neuromelioidosis and *Spirometra mansoni* infestation. Neurolisteriosis-associated brain tunneling abscess formation is rare [2,6].

Our patient developed an acute febrile illness with progressive neurological deficits over seven days. Typical clinical features of infective rhombencephalitis include fever, headache, neck stiffness, confusion, and cranial nerve palsies, all of which were observed in this case [1].

CSF analysis showed lymphocytic pleocytosis with elevated protein and a low CSF glucose level of 35.8% relative to blood glucose (Table 1). In the literature, CSF analysis in neurolisteriosis typically shows a predominant polymorphonuclear pleocytosis, with CSF glucose below 40 mg/dL in 39% of patients. CSF protein can be elevated to 100-200 mg/dL [8]. However, CSF pleocytosis and glucose levels in neurolisteriosis vary depending on the timing of lumbar puncture, prior antibiotic use, and the patient’s immune status. CSF Gram stain and culture are considered the gold standard for diagnosing listeriosis. In this patient, blood and CSF Gram stains and cultures were negative, possibly due to prior antibiotic treatment and delayed culture sampling. CSF culture positivity for *L. monocytogenes* is reported in approximately 40% of clinically suspected cases [9].

MRI of the brain demonstrated characteristic tunneling abscesses in the brainstem (Figure 2A-2E). The presence of brainstem tunneling abscesses, along with a dramatic response to IV ampicillin in an elderly, immunocompromised patient, supported the diagnosis of *Listeria *rhombencephalitis. Neuromelioidosis was excluded by performing a blood melioidosis antibody assessment.

There are seven case reports in the literature describing CNS tunneling abscess formation associated with neurolisteriosis. Only one case report has described brainstem tunneling abscess formation due to *Listeria *rhombencephalitis (Table 2).

**Table 2:** Literature review of previously reported cases of neurolisteriosis with CNS tunneling abscess formation

Author	Patient characteristics	Abscess location	*Listeria*isolation medium
Sakarunchai et al. (2016) [2]	36-year-old female on prednisolone for systemic lupus erythematosus	Left frontal lobe	Blood
Slezák et al. (2020) [6]	41-year-old immunocompetent	Cervical spine, brainstem	CSF
	50-year-old man on prednisolone for autoimmune hemolytic anemia	Right parietal lobe	Blood
	67-year-old man with multiple myeloma on chemotherapy	Right frontal lobe	Abscess biopsy tissue
Zhang et al. (2021) [10]	64-year-old female with type 2 diabetes mellitus	Left frontal and parietal lobes	Blood
Simonsen et al. (2021) [11]	39-year-old female with metastatic lung cancer on chemotherapy	Right frontal lobe and basal ganglia	Blood
Bristowe et al. (2024) [12]	82-year-old man with type 2 diabetes mellitus and monoclonal gammopathy of undetermined significance	Right frontal lobe	Blood

## Conclusions

We report a case of a 65-year-old woman with multiple myeloma undergoing chemotherapy who presented with acute CNS infection and cranial nerve palsies. MRI demonstrated the characteristic “tunnel sign” in the brain, suggestive of tunneling abscesses in the brainstem due to *Listeria* rhombencephalitis. The patient showed marked improvement following IV ampicillin therapy. This case highlights that MRI is a valuable tool for diagnosing suspected neurolisteriosis, particularly when microbiological confirmation is not available.
